# Supplementary material for: Validation of the moral foundations questionnaire-2 (MFQ-2) in Germany: Psychometric properties and associations with political ideology, religiosity, and personality
Source: PLoS One. 2026 Mar 25;21(3):e0345599. doi: 10.1371/journal.pone.0345599 (PMC13016328; doi:10.1371/journal.pone.0345599)
Supplement: S1 Appendix — The file contains: (1) demographic information; (2) the full, pre-final German version of the Moral Foundations Questionnaire-2 (MFQ-2) developed for this study; (3) self-created items to assess political ideology and (4) religiosity; (5) the German versions of the Big Five Inventory-2-Short Form (BFI-2-S); (6) the German version of the Ethics Position Questionnaire-5 (EPQ-5); (7) the AI agent detection task; and (8) the three attention check items. (DOCX) [file pone.0345599.s001.docx]

**Supplementary Information**

The supplementary information contains an overview of all measures and items of the study: (1.) demographic information; (2.) the full, pre-final German version of the Moral Foundations Questionnaire-2 (MFQ-2) developed for this study; (3.) self-created items to assess political ideology and (4.) religiosity; (5.) the German versions of the Big Five Inventory-2-Short Form (BFI-2-S); (6.) the German version of the Ethics Position Questionnaire-5 (EPQ-5); (7.) the AI agent detection task and (8.) the three attention check items. All materials are also available in German upon request.

1. Demographic information

- via PA
  - age (“What is your date of birth?”)
  - sex (“What is your sex, as recorded on legal/official documents?”)
  - first language (“What is your first language?”)
  - primary language (“What is your primary language?”)
  - current country of residence (“In what country do you currently reside?”)
  - nationality (“What is your nationality?”)
  - country of birth (“What is your country of birth?“)
  - student status (“Which of these is the highest level of education you have completed?”)
  - employment status (“What is your employment status?”)
- “In which German federal state have you spent most of your life so far?” [in political ideology section in the survey]

2. Moral Foundations Questionnaire-2 (MFQ-2; self-developed German adaptation)

[5-point-Likert scale from 1 (Strongly disagree) to 5 (Strongly agree)]

“For each of the statements below, please indicate how much you agree with it, from ‘Strongly disagree’ to ‘Strongly agree’.”

1. Caring for people who have suffered is an important virtue.
2. The world would be a better place if everyone made the same amount of money.
3. I think people who are more hardworking should end up with more money.
4. I think children should be taught to be loyal to their country.
5. I think it is important for societies to cherish their traditional values.
6. I think the human body should be treated like a temple, housing something sacred within.
7. I believe that compassion for those who are suffering is one of the most crucial virtues.
8. Our society would have fewer problems if people had the same income.
9. I think people should be rewarded in proportion to what they contribute.
10. It upsets me when people have no loyalty to their country.
11. I feel that most traditions serve a valuable function in keeping society orderly.
12. I believe chastity is an important virtue.
13. We should all care for people who are in emotional pain.
14. I believe that everyone should be given the same quantity of resources in life.
15. The effort a worker puts into a job ought to be reflected in the size of a raise they receive.
16. Everyone should love their own community.
17. I think obedience to parents is an important virtue.
18. It upsets me when people use foul language like it is nothing.
19. I am empathetic toward those people who have suffered in their lives.
20. I believe it would be ideal if everyone in society wound up with roughly the same amount of money.
21. It makes me happy when people are recognized on their merits.
22. Everyone should defend their country, if called upon.
23. We all need to learn from our elders.
24. If I found out that an acquaintance had an unusual but harmless sexual fetish I would feel uneasy about them.
25. Everyone should try to comfort people who are going through something hard.
26. When people work together toward a common goal, they should share the rewards equally, even if some worked harder on it.
27. In a fair society, those who work hard should live with higher standards of living.
28. Everyone should feel proud when a person in their community wins in an international competition.
29. I believe that one of the most important values to teach children is to have respect for authority.
30. People should try to use natural medicines rather than chemically identical human-made ones.
31. It pains me when I see someone ignoring the needs of another human being.
32. I get upset when some people have a lot more money than others in my country.
33. I feel good when I see cheaters get caught and punished.
34. I believe the strength of a sports team comes from the loyalty of its members to each other.
35. I think having a strong leader is good for society.
36. I admire people who keep their virginity until marriage.

3. Political ideology

- “When we talk about political tendencies today, we are referring to people who sympathize to varying degrees with left-wing or right-wing political views.”
  1. “Based on the political meaning of the terms ‘left’ and ‘right,’ where would you place yourself on this scale?” [10-point Likert scale from 1 (far left) to 10 (far right)]
  2. “How would you describe your political views on social issues (e.g., euthanasia, immigration)?” [continuous scale from 0 (extremely progressive) to 100 (extremely conservative)]
  3. “How would you describe your political views on economic issues (e.g., wealth distribution, taxation)?” [continuous scale from 0 (extremely progressive) to 100 (extremely conservative)]
- “Which party do you feel most affiliated with?” [categorical]
  1. CDU
  2. CSU
  3. SPD
  4. Bündnis 90 / Die Grünen
  5. FDP
  6. AfD
  7. Die Linke
  8. BSW
  9. Freie Wähler
  10. Volt
  11. Die Partei
  12. ÖDP
  13. other / not specified

4. Religiosity

- “How important is spiritual belief in your life?” [continuous scale from 0 (not important at all) to 100 (extremely important)]

5. Personality traits (Agreeableness and Extraversion) via the German version of the Big Five Inventory-2 (BFI-2-S)

[5-point-Likert scale from 1 (Strongly disagree) to 5 (Strongly agree)]

“Below you will find a series of characteristics that may apply to you. Would you say, for example, that you enjoy spending time with other people? For each of the following statements, please indicate to what extent you agree with it, from “Strongly disagree” to ‘Strongly agree’.”

1. I tend to be quiet.
2. I am compassionate, have a soft heart.
3. I am dominant, tend to act as a leader.
4. I am sometimes rude to others.
5. I am full of energy.
6. I assume the best about people.
7. I am outgoing, sociable.
8. I can be cold and uncaring.
9. I prefer to have others take charge.
10. I am respectful, treat others with respect.
11. I am less active than other people.
12. I tend to find fault with others.

6. Ethical positions via the German version of the Ethics Position Questionnaire-5 (EPQ-5)

[5-point-Likert scale from 1 (Strongly disagree) to 5 (Strongly agree)]

“Please read the following ten statements carefully and indicate whether you agree or disagree with them, ranging from ‘Strongly disagree’ to ‘Strongly agree’.”

- 1. A person should make certain that their actions never intentionally harm
     another even to a small.
  2. The existence of potential harm to others is always wrong, irrespective of the
     benefits to be gained.
  3. One should never psychologically or physically harm.
  4. One should not perform an action which might in any way threaten the dignity
     and welfare of another individual.
  5. If an action could harm an innocent other, then it should not be done.
  6. What is ethical varies from one situation and societies.
  7. Moral standards should be seen as being individualistic; what one person
     considers to be moral may be judged to be immoral by another person.
  8. Questions of what is ethical for everyone can never be resolved since what is
     moral or immoral is up to the individual.
  9. Moral standards are simply personal rules that indicate how a person should
     behave, and are not to be applied in making judgments of others.
  10. Ethical considerations in interpersonal relations are so complex that individuals
      should be allowed to formulate their own individual codes.

7. AI agent detection task

- assemble the made-up word “LUMIR” at the start of the survey using a drag-and-rank task
- Letter tiles for the word will appear on the left in a random order; the tiles should be placed correctly on the right so that the word can be read from top to bottom.

8. Attention checks

[5-point-Likert scale from 1 (Strongly disagree) to 5 (Strongly agree)]

Attention check 1 (within the first half of the MFQ-2)

- “It is important to help others in need; and if you are reading this, please select ‘Disagree’.”
- correct number of answer: 2

Attention check 2 (within the second half of the MFQ-2)

- “All people should have the same opportunities in life; and if you are reading this, please select ‘Undecided’.”
- correct number of answer: 3

Attention check 3 (within the EPQ-5)

- “Moral judgments often depend on personal context; and if you are reading this, please select ‘Strongly agree’.”
- correct number of answer: 5
