# Supplementary material for: Validation of the moral foundations questionnaire-2 (MFQ-2) in Germany: Psychometric properties and associations with political ideology, religiosity, and personality
Source: PLoS One. 2026 Mar 25;21(3):e0345599. doi: 10.1371/journal.pone.0345599 (PMC13016328; doi:10.1371/journal.pone.0345599)
Supplement: Attachment — Submitted filename: Response_to_Reviewer.docx [file pone.0345599.s002.docx]

Drs. Johannes Schwabe and Reviewer,

Thank you for your thorough and thoughtful review of our stage 1 registered report protocol *“Validation of the Moral Foundations Questionnaire-2 (MFQ-2) in Germany: Psychometric Properties and Associations with Political Ideology, Religiosity, and Personality"*. We have very carefully considered all of your and the reviewer’s concerns and suggestions and have revised our registered report protocol with your recommendations in mind. Below you will find detailed responses to each of your comments.

We truly believe that editor’s and reviewer’s feedback has made our registered report protocol much stronger. We genuinely thank the reviewer for pointing out the spots in which the previous iteration of this registered report protocol fell short in this regard.

To assist in readability, we have highlighted our revisions in the registered report protocol using track changes and noted the line numbers of the changes in our responses to the reviewer below. We hope that you can agree with our revisions and that our registered report protocol can be accepted in principle.

Thank you again for your time and service.

Sincerely,

Authors

**Editor**

Dear Dr. Hopp,

Thank you for submitting your manuscript to PLOS ONE. After careful consideration, we feel that it has merit but does not fully meet PLOS ONE’s publication criteria as it currently stands. Therefore, we invite you to submit a revised version of the manuscript that addresses the points raised during the review process.

==============================

- This is a valuable contribution to the literature, and the plan is generally sound and feasible.
- I have received one thoughtful review, and the reviewer raises a number of good points.
- Please ensure to address each of the reviewer's points in the revised protocol.
- The revised protocol may or may not be sent out for additional review after revision.

==============================

We are pleased to hear that both the editor and the reviewer see merit in our proposed study. We also thank the editor and the reviewer for highlighting points where our previous registered report protocol fell short. In addressing the reviewer’s concerns, we have clarified and revised our planned analyses in places where our previous registered report fell short.

**Reviewer 1**

The manuscript presents a Stage 1 protocol for a validation study of the Moral Foundations Questionnaire-2 (MFQ-2) in Germany. The authors describe the planned study design, questionnaire, and analysis strategy. Overall, the description of the research plan seems realistic and sensible. However, I have several suggestions for improvement that might strengthen the planned research.

We very much appreciate and thank the reviewer for their valuable and constructive criticism of our registered report protocol as well as for recognizing the feasibility of our proposed research design. By addressing each comment below, we hope the reviewer can agree that we have substantially improved our stage 1 registered report protocol.

**R1.1.** The factor structure of the MFQ-2 will be examined using exploratory and confirmatory factor analysis. It was unclear which factor analytic approach will be addressed in Hypothesis 1 (p5). It is very likely that ESEM, which allows for cross-loadings between factors, will provide a good fit to the data, whereas it might be more difficult to achieve a good fit with CFA that constrains all cross-loadings to 0. Hypothesis 1 could be more specific if, in case of ESEM, the largest size of the cross-loadings that is deemed acceptable would be specified as well.

This is an important clarification. We have revised the protocol to specify that H1a (previous H1; see R1.3.) will be primarily tested using ESEM, with CFA serving as a complementary analysis (p. 5, line 89). Regarding acceptable cross-loadings, we have now incorporated explicit thresholds into the protocol: Items will be flagged if they show cross-loadings exceeding |.30| (Hair et al., 2019) or a difference between the primary and any alternative loading of less than .20 (Howard, 2016). These criteria are outlined in the revised Structural Validation section (p. 12, lines 249–51).

**R1.2.** I was wondering if a minimal size of the validity correlations (in addition of their direction) could be formulated in Hypothesis 2 (p5).

We agree that specifying minimum effect sizes strengthens the hypotheses. We have revised H2a–c (and analogously, H3, H4a–b, H5a–b, and H6) to include effect size expectations. Based on prior MFQ-2 validation studies reporting correlations of *r* ≈ .15 to .75 (Atari et al., 2023; Zakharin & Bates, 2023), we have specified that we expect at least small-to-moderate correlations (*r* ≥ .15) in the hypothesized directions (pp. 5–6, lines 92–93, 97, 107, 110, 114–115; accordingly: p. 9, lines 183–186).

**R1.3.** The reliability of the MFQ-2 factors was not explicitly addressed in any hypothesis. I imagine that the measurement precision is an important criterion for evaluating an instrument. Therefore, it could be informative to specify an explicit hypothesis regarding reliability.

We fully agree that measurement precision is essential for evaluating an instrument. To provide a clear distinction between internal (H1a–b) and external validity (subsequent Hypotheses), we renamed the original Hypothesis 1 to H1a (p. 5, line 87; accordingly: p. 9, line 179, and p. 11, line 215) and added a new hypothesis, H1b, for reliability (p. 5 line 90–91; accordingly: p. 2, line 23, p. 5, line 85, p. 9, lines 179–180, p. 13, lines 273–275):

***H1b:*** *All six MFQ-2 subscales will demonstrate acceptable internal consistency reliability (McDonald’s ω_t_ ≥ .70).*

This minimum threshold is consistent with reliability values reported in the original MFQ-2 validation (ω = .73 to .95; Atari et al., 2023). Notably, to ensure precision, we have updated the notation from ‘ω’ to ‘ω_t_’ throughout the manuscript to explicitly refer to McDonald’s total omega, following the reporting standards of Atari et al. (2023).

**R1.4.** It was unclear why the authors aim for a sample size of 2,624 respondents, although their power analysis suggests that about 1,300 respondents would be enough (p9). Do they expect over 1,000 non-diligent respondents that need to be excluded? Otherwise, the rationale for this choice remained unclear. Also, I am unsure whether Prolific actually includes so many German participants that would be willing to participate. I had recently difficulty to recruit 1,000 individuals from Germany.

The reviewer raises a valid concern. Upon reconsideration, we agree that *N* = 2,624 was not well-justified. We have revised our recruitment target to *N* ≈ 1,200 participants. Anticipating a maximum of 15–20% exclusions due to attention checks and data quality screening, this yields a final sample of *N* ≈ 1,000 (p. 9, line 187–189; accordingly: p. 2, line 22). A recent pre-screening on Prolific indicates that 2,925 participants match our inclusion criteria and have been active within the past 90 days, ensuring the feasibility of our revised recruitment target.

We have updated the power analysis based on this revised target and our specified minimum effect size of *r* ≥ .15. These calculations confirm that a final sample of *N* ≥ 1,000 is more than sufficient for all planned analyses. Specifically, for the internal validation (H1a–b), *N* = 382 is required to detect all factor correlations with 95% power using a Bonferroni-adjusted alpha. For the correlational hypotheses (H2–H6), *N* = 571 is sufficient to detect our expected minimum effect of *r* ≥ .15 with 95% power (p. 9, lines 182–189). Furthermore, a sensitivity analysis shows that with *N* = 1,000, the study achieves 95% power to detect effects as small as *r* = .114 (p. 9, lines 189–190).

**R1.5.** I recommend dealing with missing values using multiple imputations or full maximum likelihood. Listwise deletion is generally not recommended because this can introduce additional bias in the analyses (p10).

The reviewer is correct that listwise deletion can be suboptimal. Hence, we have updated our strategy to maximize data retention. Given that all survey items are mandatory, missingness is only anticipated in cases of participant dropout or technical errors. To handle this robustly, we have implemented Full Information Maximum Likelihood (FIML) for all structural analyses (ESEM/CFA) and will utilize pairwise deletion for correlational analyses (p. 10, lines 207–210). This ensures that all available data points are utilized while providing more reliable estimates than traditional deletion methods.

**R1.6.** Given that the authors plan to modify the measurement model and allow for non-hypothesized residual correlations (p12), I strongly recommend splitting the sample into two random subsamples, identifying the optimal factor structure in the first subsample, and replicating it in the second subsample. This would inform whether the modifications are robust and generalize across samples. Also, it was unclear whether these analyses will be conducted using ESEM or CFA.

We agree that cross-validation is essential when model modifications are considered. Consequently, we have updated our analysis plan to include a split-sample procedure (p. 11, lines 215–223, p. 13, lines 264–266). The total sample (*N* ≈ 1,000) will be randomly divided into two subsamples (*n* ≈ 500 each).

Subsample 1 will serve as the calibration sample, where the factor structure will be explored and potentially refined using ESEM (addressing H1a–b). Subsample 2 will serve as the validation sample, where the final model will be tested using CFA to ensure robustness and generalizability. As our power analysis (see R1.4) indicated a minimum requirement of *N* = 382 for structural stability, each subsample is sufficiently powered for this procedure.

Regarding the reviewer’s second point, we clarified that ESEM will be our primary analytical framework due to its ability to account for minor cross-loadings, while CFA will be used in the validation step to test the final, more parsimonious structure (p. 11, lines 228–235). To maintain maximum power for the nomological network testing (H2–H6), these hypotheses will be tested using the full sample once structural validity has been established (p. 14, lines 297–298).

**R1.7.** Will CFA or ESEM be used to study the higher-order factor structure (p11)? Generally, the entire manuscript remains unclear which factor specification will be followed.

We apologize that this was unclear. We have specified the factor specifications in the revised Structural Validation section. For the first-order factor structure, ESEM will be our primary framework to account for theoretically meaningful cross-loadings, while CFA will serve as a more parsimonious complementary analysis during the validation phase (see R1.6.).

For higher-order models, we will employ traditional CFA (p. 11, lines 229, 232–234). This decision is based on the technical complexity and potential estimation problems associated with ESEM-within-CFA specifications, which are currently not considered standard practice for higher-order validation (Marsh et al., 2020).

**R1.8.** On page 10, the authors indicate to use maximum likelihood estimation because of the ordinal response format and switch to DWLS in case of convergence problems. I believe this is incorrect. The DWLS estimator is used for ordinal responses. Generally, I do not think that an ordinal CFA is necessary for seven-point response scales. These can be treated as quasi-continuous and anaylzed with ML.

The reviewer is correct, and we thank them for outlining this. Seven-point scales can indeed be treated as quasi-continuous (Rhemtulla et al., 2012), making MLR the most appropriate choice. We have, therefore, corrected the manuscript (p. 10, lines 204–206; deleted lines: p. 11, lines between 223–224) to specify MLR as the sole estimator and have removed references to ordinal CFA and the DWLS estimator.

**R1.9.** Model comparisons will be conducted by comparing information criteria (AIC, BIC) between models (p11). However, no information was provided on the size of the differences that will be considered meaningful. Also, what will be done if the two information criteria diverge in their conclusions?

We thank the reviewer for this helpful suggestion. We have added explicit criteria for model comparison to the manuscript (p. 12, lines 242–246). Following Dziak et al. (2020), we defined Δ > 10 as strong evidence for a superior model and Δ < 2 as indicating indistinguishable models. In cases of divergence between AIC and BIC or weak evidence (2 ≤ Δ ≤ 10), we will prioritize the BIC and the more parsimonious model. This strategy is specifically chosen to ensure model consistency and avoid over-parameterization in large samples (Dziak et al., 2020; Vrieze, 2012).

**R1.10.** If the goal of the present research is the development of a German version of the MFQ-2, then items must not be removed from the instrument (p12). Otherwise, it would be an adaptation, and the instrument would no longer be comparable to the original version.

We agree that maintaining the original 36-item structure is essential. We have revised the manuscript (pp. 12–13, lines 256–266) to state that the full 36-item model remains our primary focus and will be reported regardless of fit. Any item removal has been redefined as a last resort refinement, conducted only if marginal fit thresholds (CFI/TLI < .85, RMSEA > .10; see Kline, 2023) are not met. Therefore, the 36-item version remains our benchmark for cross-cultural comparability, while any modified configuration will be transparently labeled as a “German adaptation.”

**R1.11.** It was unclear how measurement invariance across the different models (configural, metric, scalar) will be evaluated (p13). Are the authors planning to rely on inference tests, descriptive comparisons of approximate fit indices, or use some effect sizes as well?

We have updated the manuscript (p. 14, lines 287–290) to clarify the evaluation of measurement invariance. To address the sensitivity of χ² difference tests in large samples (*N* ≈ 1,000), we will primarily rely on descriptive comparisons of fit indices. Following the recommendations of Chen (2007), invariance will be supported if the change in fit between increasingly restrictive models meets the criteria of ΔCFI ≥ -.010 and ΔRMSEA ≤ .015. Standard χ² difference tests will be reported as supplementary information. If full scalar invariance is not achieved, we will examine partial invariance or utilize the alignment method (Muthén & Asparouhov, 2014) to allow for valid latent mean comparisons.

**R1.12.** The Shapiro-Wilks test will definitely be significant given the large sample (p13). I do not think that the choice of the correlation coefficient should depend on the distribution of the variable. The point estimate of the Pearson correlation does not require normally distributed data, only the inference test does.

We agree with the reviewer that the Shapiro-Wilk test is likely to be oversensitive in a sample of *N* ≈ 1,000. Accordingly, we have removed the Shapiro-Wilk criterion and the conditional switch to Spearman’s ρ from the analysis plan (p. 14, lines 299–303). We will use Pearson’s *r* as the standard correlation coefficient throughout. As the reviewer notes, the point estimate of *r* does not require normality, and the Central Limit Theorem ensures that the sampling distribution for inference tests will be approximately normal at this sample size (Bishara & Hittner, 2012). To ensure transparency, we will report skewness and kurtosis values and use visual inspection (Q-Q plots) to identify any extreme distributional anomalies.

**R1.13.** Hypothesis 3 is best analyzed as a moderated regression, rather than t-test on a subsample (p13). This avoids dichotomizing a metric variable and allows analyzing the entire sample without reverting to subgroup analyses.

We agree with this methodological recommendation. We have revised our approach (p. 15, lines 310–315; accordingly: lines 331–333; see also R1.2. and p. 6, lines 105–107): H3 will now be tested using a linear regression on the full sample. Following the logic of a moderated analysis, we will use a difference score (individualizing minus binding foundations) as the dependent variable and political ideology as a continuous predictor. A significant negative regression coefficient—assuming higher values indicate conservatism—would support H3, indicating that increasing conservatism is associated with a decrease in the prioritization of individualizing over binding foundations.

**R1.14.** I also recommend examining the construct validity (p14) using multiple regression for all variables simultaneously as this can accommodate metric and categorical variables, without the need for an artificial dichotomization of metric variables.

We appreciate this suggestion for strengthening the construct validity analyses. Multiple regression is a robust way to account for shared variance among predictors. We have revised the manuscript (p. 16, lines 334–340) to include supplementary multiple regression analyses. While our primary hypothesis tests (H2–H6) will continue to rely on bivariate correlations to ensure comparability with the existing MFQ-2 validation literature (Atari et al., 2023; Frisari et al., 2025), the additional regressions will allow us to examine the unique contributions of political ideology and religiosity, personality traits, and demographic variables (gender, age) while controlling for one another (following Zakharin & Bates, 2023). This approach avoids artificial dichotomization and provides a more nuanced view of the moral foundations’ nomological network.

**References**

Atari, M., Haidt, J., Graham, J., Koleva, S., Stevens, S. T., & Dehghani, M. (2023). Morality beyond the WEIRD: How the nomological network of morality varies across cultures. *Journal of Personality and Social Psychology*, *125*(5), 1157–1188. https://doi.org/10.1037/pspp0000470

Bishara, A. J., & Hittner, J. B. (2012). Testing the significance of a correlation with nonnormal data: Comparison of Pearson, Spearman, transformation, and resampling approaches. *Psychological Methods*, *17*(3), 399–417. https://doi.org/10.1037/a0028087

Chen, F. F. (2007). Sensitivity of Goodness of Fit Indexes to Lack of Measurement Invariance. *Structural Equation Modeling: A Multidisciplinary Journal*, *14*(3), 464–504. https://doi.org/10.1080/10705510701301834

Dziak, J. J., Coffman, D. L., Lanza, S. T., Li, R., & Jermiin, L. S. (2020). Sensitivity and specificity of information criteria. *Briefings in Bioinformatics*, *21*(2), 553–565. https://doi.org/10.1093/bib/bbz016

Frisari, F. V., Bonora, M., Parisse, C., Livi, S., & Kosic, A. (2025). Italian adaptation and psychometric analysis of the moral foundations questionnaire 2. *Current Psychology*, *44*(4), 2237–2246. https://doi.org/10.1007/s12144-025-07310-3

Hair, J. F., Black, W. C., Babin, B. J., & Anderson, R. E. (2019). *Multivariate data analysis* (Eighth edition). Cengage.

Howard, M. C. (2016). A Review of Exploratory Factor Analysis Decisions and Overview of Current Practices: What We Are Doing and How Can We Improve? *International Journal of Human–Computer Interaction*, *32*(1), 51–62. https://doi.org/10.1080/10447318.2015.1087664

Kline, R. B. (2023). *Principles and Practice of Structural Equation Modeling*. Guilford Publications.

MacCallum, R. C., Widaman, K. F., Zhang, S., & Hong, S. (1999). Sample size in factor analysis. *Psychological Methods*, *4*(1), 84–99. https://doi.org/10.1037/1082-989X.4.1.84

Marsh, H. W., Guo, J., Dicke, T., Parker, P. D., & Craven, R. G. (2020). Confirmatory Factor Analysis (CFA), Exploratory Structural Equation Modeling (ESEM), and Set-ESEM: Optimal Balance Between Goodness of Fit and Parsimony. *Multivariate Behavioral Research*, *55*(1), 102–119. https://doi.org/10.1080/00273171.2019.1602503

Muthén, B., & Asparouhov, T. (2014). IRT studies of many groups: The alignment method. *Frontiers in Psychology*, *5*. https://doi.org/10.3389/fpsyg.2014.00978

Rhemtulla, M., Brosseau-Liard, P. É., & Savalei, V. (2012). When can categorical variables be treated as continuous? A comparison of robust continuous and categorical SEM estimation methods under suboptimal conditions. *Psychological Methods*, *17*(3), 354–373. https://doi.org/10.1037/a0029315

Vrieze, S. I. (2012). Model selection and psychological theory: A discussion of the differences between the Akaike information criterion (AIC) and the Bayesian information criterion (BIC). *Psychological Methods*, *17*(2), 228–243. https://doi.org/10.1037/a0027127

Zakharin, M., & Bates, T. C. (2023). Moral Foundations Theory: Validation and replication of the MFQ-2. *Personality and Individual Differences*, *214*, 112339. https://doi.org/10.1016/j.paid.2023.112339
